# Irreversible and quantum thermodynamic considerations on the quantum zeno effect

**DOI:** 10.1038/s41598-023-38040-w

**Published:** 2023-07-04

**Authors:** Umberto Lucia

**Affiliations:** 1 Dipartimento Energia “Galileo Ferraris”, Politecnico di Torino, Corso Duca degli Abruzzi 24, 10129 Torin, Italy; 2 INFN-Sezione di Torino, Turin, Italy

**Keywords:** Thermodynamics, Quantum mechanics, Quantum information

## Abstract

The quantum zeno effect slows down the quantum system’s time evolution under frequent measurements. This paper aims to study this quantum effect by introducing the definition of time based on an irreversible thermodynamic analysis of quantum systems. Consequently, the quantum zeno effect requires (i) high values of the electromagnetic entropy generation rate related to the spontaneously down-converted light and (ii) a decrease in the quantum system’s entropy value. So, the quantum zeno effect is a quantum process related to the interaction between a quantum system and the electromagnetic waves of the measurement device, causing a quantum thermodynamic stationary state. Last, the fundamental role of irreversibility emerges.

## Introduction

The quantum zeno effect, also known as Turing’s paradox, is a feature of quantum mechanical systems that allows the time evolution of a particle to be slowed down by measuring it frequently enough against a chosen measurement setting^1^.

Alan Turing was the first to mention this phenomenon in his private correspondence with his colleague Robin Grandy dated 1954^2^: Turing pointed out this paradoxical result. Frequent measurements can slow the evolution of a quantum system, hindering transitions to states different from the initial one^3^. This phenomenon is considered related to the quadratic behaviour of the survival probability at short times for the Schrödinger equation^4^, as pointed out by John von Neumann^5^. Degasperis, Fonda and Ghirardi^6^ carried out the first approach to the phenomenon, while Baidyanath Misra and George Sudarshan^1^ developed its rigorous description.

In 1988, Cook^7^ proposed an experimental approach, based on oscillating systems, to verify the quantum zeno effect, then carried out by Itano et al.^8^. Then, other experimental confirmations of the phenomenon were obtained in different physical contexts, such as photon polarization^9^, nuclear spin isomers^10^, optical pumping^11^, etc. In the analysis of the experiment carried out by Itano et al.^8^, Venugopalan and Ghosh^12^ showed that the experimental results are related to the environment-induced decoherence theory^13^, for which the environment of a quantum system can monitor some of the system’s observables, with the result that the eigenstates of those observables are continuously subjected to decoherence, showing classical-like states. Indeed, they highlighted that, during the measurement process, the quantum system is coupled to its external environment, which leads to decoherence over a characteristic time scale, named *decoherence time*. In the context of quantum zeno effect, Facchi and Pascazio^14^ highlight that a down-conversion process in a non-linear crystal can be studied as the decay of a pump photon into a pair of photons of lower frequency, and the energy of the spontaneously down-converted light monotonously increases. Moreover, in 2006, Streed et al. observed experimentally the dependence of the quantum zeno effect on measurement pulse characteristics^15^.

Today, the growing interest in the quantum zeno effect is related to its theoretical implication in the fundamentals of quantum mechanics, and also in its applications to spin polarization in gases^16^, control of decoherence in quantum computing^17^, etc. The physical meaning of decoherence has been clarified by means of quantum and classical irreversibility^18^, highlighting that decoherence is an irreversible process^19^, due to interaction between the quantum system and its environment.

The Second Law of Thermodynamics states that the entropy of an isolated system increases with time (for irreversible processes) or remains constant^20^. Remembering that measurement on a quantum system is an irreversible process^5,21^, the entropy is expected to increase, while on the contrary, the information obtained by frequent observation on a quantum system has been proven to decrease the entropy of the system itself^22^.

Recently, a thermodynamic approach^23–25^ to irreversibility in quantum systems has been developed based on the continuous interaction between the environmental electromagnetic waves and the matter, analysing the absorption-emission of a photon by an atomic electron, obtaining a thermophysical model of quantum thermodynamics in agreement with the experimental results^26–29^. In this context, quantum system is considered as an open system, due to the photon inflow and outflow.

This approach can be extended to the quantum zeno effect, considering the coupled quantum system-experimental device as the isolated system while the quantum system and the device are independently two open subsystems^30^.

This paper aims to develop this thermophysical analysis of the quantum zeno effect, pointing out the fundamental role of irreversibility and its relation with the definition of time.

## Results

The result of this paper are Eqs. (7) and (11), with respect to which we can highlight:

- The interaction between a quantum system and a measurement device can be studied as the interaction between a quantum system and a photon. Following Einstein, this interaction is irreversible, and concerning it, we have introduced the definition of the time interval for which the quantum system evolves. So, the unitary evolution operator results related to this time interval definition;
- The quantum zeno effects requires that the eigenvalue of the unitary operator results in 1. This can be obtained by high values of electromagnetic entropy generation rate, $\Sigma$, which is related to the spontaneously down-converted light, following the physical evidence summarised in Ref.^14^;
- The quantum zeno effect requires small values of a time interval. This implies a high electromagnetic entropy generation rate, $\Sigma$, but also small values of entropy generation, $\sigma$, which means that the quantum system decreases its entropy value, in agreement with the experimental evidence as reported in Ref.^22^
- When the rate $\sigma /\Sigma$ is not small, the eigenvalue of the unitary evolution operator does not result in 1, so the evolution inhibition does not occur, and the Quantum anti-Zeno Effect emerges^31^.

These considerations lead to the state that the quantum zeno effect is a quantum process related to the interaction between a quantum system and the electromagnetic waves of the measurement device, such that the result is a quantum thermodynamic stationary state ($dS/dt = 0$, with *S* entropy of the control volume composed by the quantum system and the measurement device). Indeed, considering the thermodynamic universe composed by the quantum system and the measurement device, this effect results in a dynamical process in which the system exchanges photons inflow and outflow such that the entropy generation rate $\Sigma$ emerges from the entropy balance for the quantum system, as follows:1where $\dot{n}_{ph}$ is the photon flux, *s* is the entropy generated by the interaction with each photon, *in* and *out* mean inflow and outflow respectively. Consequently, the total entropy of the thermodynamic universe results constant ($dS = 0$), such that:

- The device (environment) increases its entropy due to the entropy generation rate, $\Sigma$, produced by the electromagnetic waves interaction;
- The quantum system reduces its entropy; indeed, its entropy variation results $\Delta S_{sys} = \Delta S - \int _0^\tau \Sigma \, dt = -\sigma$, where $S=- \text {Tr}(\hat{\rho }\,\ln (\hat{\rho })) = -\sum _n\,w_n\,\ln (w_n)$, where﻿ $\hat{\rho}$ is the density operator, Tr is the trace over a complete set of states and $w_n$ denotes the probability of such a state $\left| n_i\right\rangle$^32,33^.

All these considerations allow us to point out that the thermodynamic approach suggested can describe and explain the quantum systems’ behaviour concerning the quantum zeno effect.

## Discussion

The quantum zeno effect is a quantum-mechanical process that slows down a quantum system’s time evolution measuring it frequently enough for some chosen measurement setting^1^. The effect consists in freezing the evolution of a quantum system by measuring it frequently enough in its known initial state. Recently, the quantum zeno effect definition has been extended considering it as the suppression of unitary time evolution in quantum systems provided by a variety of interactions^16,34^. It appears only in systems with distinguishable quantum states, so it cannot be applied in classical system and macroscopic bodies^35^.

The study of this effect has pointed out that application of a series of strong and fast perturbations of a quantum system can decouple it from its *decohering* environment^36^. Thus, frequent measurements can inhibit decay of a system, while measurements applied more slowly can enhance decay rates (Quantum anti-Zeno Effect)^31^.

In this context, the transitions from the subspace without decoherent loss of a qubit to a state with a qubit lost in a quantum computer are particularly interesting for application to quantum computer and physics of computation^37^; indeed, for the qubit correction, it is sufficient to determine whether the decoherence has already occurred or not^37^.

During periodical measurements, with a finite interval of time $\tau$, at each measurement, the wave function collapses to an eigenstate of the measurement operator. Then, among the measurements, the system evolves into a superposition of states. When this superposition state is measured, it collapses with a probability proportional to $\tau ^2$, so for very short time intervals, the probability of collapse back to the initial state becomes around 1^38^. Following the decoherence theory, the collapsing time is related to the decoherence time of the system coupled to the environment with thermal noise. The stronger the coupling is, the shorter the decoherence time is, and the faster it will collapse^39^.

The thermodynamic approach allows us to address answers to the experimental evidences by involving time definition based on the evaluation of the irreversibility. So, the fundamental role of irreversibility^30^ emerges also in quantum zeno effect. Particularly interesting are the thermodynamic considerations of quantum computing. Indeed, it is accepted that quantum computing is in principle reversible: an ideal quantum algorithm consists in initializing the data register in a state of the computational basis, followed by a unitary operation, and a final measurement in the computational basis. There is no back-action associated with this final measurement, so there is no heat dissipation associated with the reset. But, any single gate activation requires 10^−21^ J of energy, which is the same order of magnitude as the heat dissipated by the erasure of the single bit. These quantum processes generate entropy production, so quantum computing can be considered reversible, but quantum circuits, i.e., quantum computers, are irreversible. Their irreversibility leads to decoherence, and the quantum zeno effect has been shown to play a fundamental role in the control of the decoherence in the analysis of the Josephson junction in superconducting qubits^4^.

This new frontier of thermodynamics could represent a topic of investigation for the optimisation of quantum circuits. Indeed, the quantum zeno effect has attracted interest in the thermodynamic analysis of quantum information. In the context of information, entropy fluctuations represent a very interesting topic: Ahmadi et al.^40^ have analysed the stochastic quantum processes due to quantum correlations concerning also memory effects concerning the apparent violation of the second law of thermodynamics. They extend the second law to quantum processes by incorporating information explicitly into this thermodynamic law, by providing the meaning of flows and backflows of information, proving that quantum thermodynamic force is responsible for encoding and decoding information even when a feedback controller outside the system is involved in the process.

## Methods

The effect of a measure on a quantum system^41^, from a mathematical viewpoint, consists in the collapse of its full wave function $\left| \psi \right\rangle$ into an eigenstate, $\left| \psi _i\right\rangle$, of the state bases of Hilbert space $\mathcal {H}$, i.e.,^42^:

2 $$\begin{aligned} {} & \left\langle \psi _i |\psi _i\right\rangle = 1 \\&\left\langle \psi _i |\psi _j\right\rangle = 0, \forall j\ne i \end{aligned}$$

where $\left| \psi _j\right\rangle$ are the states in the Hilbert space $\mathcal {H}$ and $\left| \psi \right\rangle$ is the quantum pure state such that^43^:

3 $$\begin{aligned} \left| \psi \right\rangle \ne \left| \psi _1\right\rangle \otimes \left| \psi _2\right\rangle \otimes \dots \otimes \left| \psi _n\right\rangle \end{aligned}$$

where $\otimes$ is the tensorial product.

The time evolution of the interaction is described by the Schrödinger equation^42^:

4 $$\begin{aligned} H\,\left| \psi (t)\right\rangle = -i\hslash \,\frac{\partial \psi }{\partial t} \end{aligned}$$

where *H* is the Hamiltonian operator, $\hslash = h/2\pi$ with $h = 6.62607004 \times 10^{-34}$ J s is the Planck constant, *t* is the time and $i = \sqrt{-1}$. If the Hamiltonian is independent of time, then the unitary time evolution operator *U*(*t*) can be introduced, obtaining^42^:

5 $$\begin{aligned} \left| \phi (\tau )\right\rangle = U(\tau )\,\left| \psi (0)\right\rangle = e^{-i\,H\,\tau /\hslash }\,\left| \psi (0)\right\rangle \end{aligned}$$

where $\left| \psi (0)\right\rangle$ is the state function evaluated in $t=0$, the initial state, and $\left| \phi (\tau )\right\rangle$ is the state function evaluated in $t = \tau$, with $\tau$ time interval.

Consequently, the quantum zeno effect can be analysed by considering the state function evolution as follows:

6 $$\begin{aligned} \left| \phi (\tau )\right\rangle = e^{-i\,H\,\tau /\hslash }\,\left| \psi (0)\right\rangle = \left| \psi (0)\right\rangle \end{aligned}$$

which allows us to focus our study on the definition of time interval $\tau$.

Indeed, recently, a thermodynamic approach to time interval definition has been developed^44–46^ about the analysis of irreversibility^23,23,24^ in photon-atomic-electron interaction^26–30^.

In this approach, time is conjectured to be related both to the entropy production (also called entropy generation in engineering thermodynamics) and to the entropy production rate (also called entropy generation rate in engineering thermodynamics), in agreement with the approach of Planck and Einstein, who highlighted that the law of system evolution consists of the law of entropy evolution^47^.

The physic bases of the thermodynamic approach are the followings:

- The atom, without interaction, can be considered an isolated system, and any process inside it is completely reversible;
- The atom, in interaction with a photon, is an open system where inflow and outflow of photons can occur;
- The atom in interaction is subjected to an irreversible process due to the perturbation of its center of mass: the irreversibility emerges as interaction with the environment;
- During the absorption-emission phenomena, electrons seem to follow a reversible energetic pathway^48–50^ (Franck-Condon approximation), because considering a single atom or molecule, the energy perturbation of the center of mass results of the order of 10^−13^ J, small compared to the electron transition energy which is of the order of 10^−8^ J, with a related excited state lifetime of the order of 10^−8^ s^50^. But, the reversible atom is only an approximation^48–50^, that cannot be introduced in the study of irreversibility, due to the need to consider all the phenomena of the system photon-atom-environment^51^, during the photon-atomic electron interaction, following some recent experimental results^52^;
- The consequence of the interaction between the bound electron and the photon is an entropic footprint in the quantum system.

Similarly to rational mechanics, where position and velocity can be used as independent variables for the state space, we use the entropy production $\sigma$ and the entropy production rate $\Sigma$ as independent variables of the state space $\Omega =\{(\sigma ,\Sigma )\}$^44,46^. In this context, some comments on entropy must be included. Indeed, different analytical forms of entropy have been introduced in thermodynamics, statistical physics and information sciences^53–56^. The entropy variation $\Delta S$ is a footprint of the change of the state of the system, during its evolution between its initial and final states^54,55^. Different approaches have been developed to derive an irreversible description of processes^57–59^. An interesting approach to irreversible processes^53,60,61^ is to evaluate $\sigma = \sum _{i}J_{i}X_{i} > 0$, where $J_{i}$ are flows of the matter, heat, etc., and $X_{i}$ are generalized driving forces for vector transport processes or for chemical reactions, etc. In this paper, this approach is interesting in relation to the study of Ahmadi et al. in relation to information and entropy fluctuation, because it is based on the definition of thermodynamic forces. The $J_{i}$ and $X_{i}$ are linearly related when the system is not too far from equilibrium. Consequently, $J_{i} = \sum _{i}L_{ij}X_{j}$, where the $L_{ij}$ are called phenomenological transport coefficients. The aim of the nonequilibrium statistical mechanics is to obtain the macroscopic thermodynamic laws from microscopic time reversible dynamics^53–55,60–64^, still under intense studies from various viewpoint^65^. Irreversibility emerges from the interaction between systems and their environment. In the analysis of the quantum zeno effect, the interconnection between macroscopic approach to irreversibility and microscopic behaviour of the systems must be considered in the context of its temporal evolution. This analysis is based on the recent thorough study^54,55^ of the concept of time, and on the definition of time^66^. The fundamental assumption is that time may be defined by means of the entropy production and the entropy rate, both due to irreversibility, and considered as independent variables as usually done for the generalised coordinates and the generalised velocities in Rational Mechanics^67^. Consequently, a possibility to design a kind of *thermodynamic clock*^66^, by using certain properties of a black body, has been introduced. Some example of applications of this approach are shown in Ref.^46^.

Moreover, radiative processes in matter and its link to entropy variation are well known processes^68–74^ in non-equilibrium thermodynamics. A recent analysis of irreversibility concerning the electromagnetic interaction with atoms and molecules, has highlighted that the continuous photon-electron interaction due to the environmental electromagnetic fields, due to the continuous thermal nonequilibrium, represents an energy footprint in the environments^23,66^, in agreement with the experimental results on the irreversibility of the electromagnetic interaction with atoms and molecules, developed in the late sixties. Here, we use these results. A deep analysis of this thermodynamic approach is developed in Ref.^46^, while a deep discussion on entropy and electromagnetic fields is developed in Ref.^75^.

Consequently, the thermodynamic definition of time interval, $\tau$, has been introduced as^25,44,46,46^:

7 $$\begin{aligned} \tau = \frac{\sigma }{\Sigma } \end{aligned}$$

where the entropy production rate $\Sigma$ results^75^:

8 $$\begin{aligned} T_0 \,\Sigma = \int_V \bigg(\frac{1}{2}\varepsilon _0 c E_{el}^2 + \frac{1}{2\mu _0} c B_m^2\bigg) dV \approx \frac{V}{2}\varepsilon _0 c E_{el}^2 + \frac{V}{2\mu _0} c B_m^2 \end{aligned}$$

where $E_{el}$ is the electric field, $B_m$ is the magnetic field, $c = 299 792 458$ m s^−1^ is the speed of light, $\varepsilon _0 = 8.8541878128(13)\times 10^{-12}$ F m^−1^ is the electric permittivity in vacuum and $\mu _0 = 4\pi \times 10^{-7}$ H m^−1^ is the magnetic permeability in vacuum, *A* is the area of the border of the thermodynamic control volume, and $T_0$ is the environmental temperature, while the entropy production has been evaluated with respect to the semi-classical analysis of the photon-bound electron interaction^23^:

9 $$\begin{aligned} T_0 \sigma = \frac{m_e}{M}\, E_\gamma \end{aligned}$$

where $T_0$ is the environmental temperature, and $E_\gamma$ is the energy of the photon. So, the time interval has been evaluated by means of measurable physical quantities as follows:

10 $$\begin{aligned} \tau =\frac{2\,m_e}{V\,M\,c}\,\frac{E_\gamma }{\varepsilon _0\, E_{el}^2 + \mu _0^{-1} \, B_m^2} \end{aligned}$$

As a consequence of this definition the unitary operator evaluated in the time $t = \tau$ results:

11 $$\begin{aligned} U(\tau ) = \exp \bigg (-\frac{i}{\hslash }\,H\,\frac{\sigma }{\Sigma }\bigg ) \end{aligned}$$

## Data Availability

All data generated or analysed during this study are included in this published article.
